# Clinical performance comparators in audit and feedback: a review of theory and evidence

**DOI:** 10.1186/s13012-019-0887-1

**Published:** 2019-04-24

**Authors:** Wouter T. Gude, Benjamin Brown, Sabine N. van der Veer, Heather L. Colquhoun, Noah M. Ivers, Jamie C. Brehaut, Zach Landis-Lewis, Christopher J. Armitage, Nicolette F. de Keizer, Niels Peek

**Affiliations:** 1 Department of Medical Informatics, Amsterdam UMC, Amsterdam Public Health Research Institute, University of Amsterdam, Amsterdam, The Netherlands; 2 NIHR Greater Manchester Primary Care Patient Safety Translational Research Centre, Manchester Academic Health Science Centre, The University of Manchester, Manchester, UK; 3 Centre for Health Informatics, Division of Informatics, Imaging and Data Sciences, Faculty of Biology, Medicine and Health, Manchester Academic Health Science Centre, The University of Manchester, Manchester, UK; 4 Occupational Science and Occupational Therapy, University of Toronto, Toronto, Ontario Canada; 5 Family and Community Medicine, Women’s College Hospital, University of Toronto, Toronto, Ontario Canada; 6 Clinical Epidemiology Program, Ottawa Hospital Research Institute, Ottawa, Ontario Canada; 7 School of Epidemiology and Public Health, University of Ottawa, Ottawa, Ontario Canada; 8 Center for Health Informatics for the Underserved, Department of Biomedical Informatics, University of Pittsburgh, Pittsburgh, PA USA; 9 Manchester Centre for Health Psychology, Division of Psychology and Mental Health, The University of Manchester, Manchester, UK; 10 NIHR Manchester Biomedical Research Centre, Manchester Academic Health Science Centre, The University of Manchester, Manchester, UK

**Keywords:** Benchmarking, Medical audit, Feedback, Quality improvement

## Abstract

**Background:**

Audit and feedback (A&F) is a common quality improvement strategy with highly variable effects on patient care. It is unclear how A&F effectiveness can be maximised. Since the core mechanism of action of A&F depends on drawing attention to a discrepancy between actual and desired performance, we aimed to understand current and best practices in the choice of performance comparator.

**Methods:**

We described current choices for performance comparators by conducting a secondary review of randomised trials of A&F interventions and identifying the associated mechanisms that might have implications for effective A&F by reviewing theories and empirical studies from a recent qualitative evidence synthesis.

**Results:**

We found across 146 trials that feedback recipients’ performance was most frequently compared against the performance of others (benchmarks; 60.3%). Other comparators included recipients’ own performance over time (trends; 9.6%) and target standards (explicit targets; 11.0%), and 13% of trials used a combination of these options. In studies featuring benchmarks, 42% compared against mean performance. Eight (5.5%) trials provided a rationale for using a specific comparator. We distilled mechanisms of each comparator from 12 behavioural theories, 5 randomised trials, and 42 qualitative A&F studies.

**Conclusion:**

Clinical performance comparators in published literature were poorly informed by theory and did not explicitly account for mechanisms reported in qualitative studies. Based on our review, we argue that there is considerable opportunity to improve the design of performance comparators by (1) providing tailored comparisons rather than benchmarking everyone against the mean, (2) limiting the amount of comparators being displayed while providing more comparative information upon request to balance the feedback’s credibility and actionability, (3) providing performance trends but not trends alone, and (4) encouraging feedback recipients to set personal, explicit targets guided by relevant information.

**Electronic supplementary material:**

The online version of this article (10.1186/s13012-019-0887-1) contains supplementary material, which is available to authorized users.

## Introduction

Audit and feedback (A&F), a summary of clinical performance over a specified period of time, is one of the most widely applied quality improvement interventions in medical practice. A&F appears to be the most successful if provided by a supervisor or colleague, more than once, both verbally and written, if baseline performance is low, and if it includes explicit targets and an action plan [1, 2]. However, reported effects vary greatly across studies and little is known about how to enhance its effectiveness [3]. In order to advance the science of A&F, the field has called for theory-informed research on how to best design and deliver A&F interventions [4, 5]. Numerous hypotheses and knowledge gaps have been proposed requiring further research to address outstanding uncertainty [5, 6]. One area of uncertainty is the choice of performance comparator included in feedback reports.

Although it is feasible to provide clinical performance feedback without an explicit comparison [7, 8], feedback is typically provided in the context of a performance comparator: a standard or benchmark to which the recipient’s observed performance level can be compared. Comparators play an important role in helping feedback recipients to identify discrepancies between current and desirable practice [9] and improve self-assessments [10]. While most often performance is compared against the average of a peer group [11], many other potential comparators have been proposed in the literature. The choice of comparator may have important implications for what message is conveyed by the feedback, and therefore how recipients react to it [12]. For instance, if a physician’s performance level has improved since the previous audit but remains well below national average, comparing against the physician’s previous level would suggest that there is no need for change, whereas comparing against the national average would suggest the opposite. At the same time, existing psychological theories suggest that the mechanisms by which recipients respond to feedback are complex, making it less obvious that recipients adopt an ‘externally imposed’ performance comparator as a personal target [7, 13]. Empirical studies show that, instead, recipients may reject feedback recommendations to pursue other levels of performance [14, 15]. To date, little evidence informs A&F intervention designers about which comparators should be chosen under what circumstances and how they should be delivered to the recipients [5, 16].

We aim to inform choices regarding performance comparators in A&F interventions and help identify causal mechanisms for change. Our objective was to (1) describe choices for delivering clinical performance comparators in published A&F interventions and (2) identify the associated mechanisms from theories and empirical studies that might have implications for effective A&F.

## Methods

To identify current choices for performance comparators, we examined all A&F interventions evaluated in the 146 unique trials included in the 2012 Cochrane review [1] and the 2017 systematic review of electronic A&F [2]. The Cochrane review spanned 1982–2011; the systematic review spanned 2006–2016. Both reviews included the databases Cochrane Central Register of Controlled Trials, MEDLINE, EMBASE, and CINAHL. We developed a data extraction sheet and guide in order to extract details about delivered comparators from all included studies. These details included what comparators were delivered, their origin, specific values delivered, and the rationale for their use. The guide and sheet were piloted by 2 reviewers (WG and BB) on 10 initial studies followed by a second pilot on 10 additional studies, each after which improvements to terms and definitions were made. WG and BB independently extracted the data; disagreements were resolved through discussion.

To identify the potential mechanisms associated with each of the different comparators that have implications for effective A&F, we reviewed existing behaviour change theories and evidence from empirical A&F studies . Candidate theories were identified from a systematic review of theories used in randomised trials of A&F [17], contact with experts, and a supplemental theory-focused literature search following the methodology detailed by Booth and Carroll [18](Additional files 1). Empirical studies were the randomised trials included in the two reviews [1, 2], and qualitative evaluation studies included in the systematic review and meta-synthesis that was recently undertaken by part of the study team [19]. We included theories and empirical studies if they described explanations of why, how, or when a behaviour may or may not occur as a result of the comparator choice within the context of receiving clinical performance feedback. From the included theories and randomised trials, we summarised relevant predictions and evidence. From the qualitative studies, we extracted and coded excerpts in batches using Framework Analysis [20] and Realistic Evaluation [21, 22] (see details in [19]). We used an iterative process to formulate mechanisms for each comparator and refine and generalise across the included theories and empirical studies [23, 24].

The consolidated results were discussed, refined, and agreed with the team. The 10-member study team has extensive expertise in designing and evaluating A&F interventions, behaviour change, implementation science, and health psychology. Three authors (HC, NI, JB) previously reviewed or have been involved in reviewing 140 randomised A&F trials [1, 11], 3 authors (BB, SvdV, NP) reviewed 7 randomised trials of electronic A&F [2], and 4 authors (WG, BB, SvdV, NP) have reviewed 65 qualitative studies of A&F [19]. The team also included clinicians and experience as feedback recipient or feedback designer.

In the ‘Results’ section, we presented the descriptions and frequency with which performance comparators have been used in randomised trials of A&F interventions, followed by the comparators’ mechanisms supported by theory and empirical evidence.

## Results

Table 1 summarises the key characteristics of the included 146 RCTs [1, 2] and 65 qualitative evaluation studies [19] of A&F interventions. We found that 98 of the 146 (67.1%) included A&F interventions used performance comparators within feedback messages; the remaining 48 intervention trials either explicitly stated they did not use a comparator or did not mention it. Possible comparators included the performance achieved by other health professionals (benchmarks, *n* = 88; 60.3%), recipients’ own historical performance (trends, *n* = 17; 9.6%), or target standards (explicit targets, *n* = 16; 11.0%). Several interventions used more than 1 type of comparator (*n* = 19; 13.0%). Only 8 (5.5%) trials reported a rationale for using their specified comparator. We included 12 theories relating to general feedback mechanisms [7, 9, 25], goal-setting [13], guideline adherence [26], psychology [27–30], and sociology [31–33], and incorporated empirical findings from 5 randomised controlled trials and 42 qualitative studies to help explain comparator mechanisms and their potential effects on clinical performance. Table 2 provides these mechanisms and their theoretical and empirical support. Table 3 shows the details and frequencies of the comparators delivered in A&F interventions.

**Table 1:** Study characteristics

Characteristic	Randomised controlled trials (*n* = 146); *n* (%)	Qualitative studies (*n* = 65); *n* (%)
Publication date		
2012–2016	2 (1)	42 (65)
2006–2011	36 (25)	15 (23)
1996–2005	76 (52)	8 (12)
1986–1995	20 (14)	–
Before 1986	12 (8)	–
Risk of bias		
Low risk	47 (32)	9 (14)
Moderate/unclear	73 (50)	47 (72)
High	26 (18)	9 (14)
Continent		
North America	82 (56)	22 (34)
Europe	46 (32)	37 (57)
Australia	11 (8)	2 (3)
Africa	2 (1)	2 (3)
Asia	4 (2)	0 (0)
South America	0 (0)	2 (3)
Clinical setting		
Outpatient	99 (68)	31 (48)
Inpatient	37 (25)	30 (46)
Other/unclear	10 (7)	4 (7)
Clinical topic		
Diabetes/cardiovascular disease management	32 (22)	20 (31)
Laboratory testing/radiology	21 (14)	0 (0)
Prescribing	33 (23)	11 (17)
Other (e.g. preventive care, nursing, surgery)	52 (36)	34 (52)

**Table 2:** Potential mechanisms and effects of clinical performance comparators and their theoretical and empirical support

Comparator	Potential mechanisms and effects	Theoretical and empirical support
Benchmarks	Increases feedback effectiveness by reducing complexity (enabling comparison with others enables recipients to better understand how well they are performing and which areas require improvement) and increasing social influence (by harnessing competition between recipients, changing recipients’ behaviour if they see others behaving differently, and trying to maintain their status in a group of high performing clinicians).	Theories (*n* = 4): Social Comparison Theory [31], Persuasion Theory [27], Social Norms Theory [33], Reference Group Theory [32]. Qualitative studies (*n* = 12): [34–52]. RCTs (*n* = 2): [53, 54]
	Debilitates feedback effectiveness by directing attention away from the performance task at hand (e.g. prescribing appropriate medication) which allows recipients to explain away potentially bad performance if overall performance is low.	Theories (*n* = 1): Feedback Intervention Theory [7]
	Induces both positive and negative emotions dependent on whether relative performance level is high or low respectively by increasing competition through social influence.	Theories (*n* = 1): Social Comparison Theory [31]. Qualitative studies (*n* = 7): [39, 49, 55–59].
	Benchmarking against a *reference group* considered irrelevant or unfair by recipients (e.g. due to case-mix difference or inadequate statistical adjustment in outcome measures) inhibits feedback acceptance by decreasing credibility and perceived validity.	Theories (*n* = 1): Reference Group Theory [32]. Qualitative studies (*n* = 8): [36, 39, 40, 51, 52, 61–63].
	Benchmarking against *values* that reflect mean or median performance inhibits action by limiting recipients’ perception of room for improvement (e.g. comparing against the mean only demonstrates discrepancies to half of recipients).	Theories (*n* = 2): Control Theory [9], Goal-setting Theory [13]. Qualitative studies (*n* = 3): [35, 59, 68]. RCTs (*n* = 3): [65–67].
	Benchmarking against *values* (e.g. the 90th percentile) inhibit feedback acceptance by low performers if they consider the discrepancy too large and unachievable.	Theories (*n* = 1): Goal-setting Theory [13]. Qualitative studies (*n* = 2): [35, 62].
	Benchmarking against *identifiable individual peers* may increase effectiveness because recipients can choose the most relevant peers for comparison and increases their sense of competition knowing that their own performance is reported to others.	Theories (*n* = 2): Social Comparison Theory [31], Reference Group Theory [32].
	Benchmarking against *identifiable individual peers* inhibits feedback acceptance when recipients consider (semi)public reporting of their own performance inappropriate and a threat to their autonomy.	Qualitative studies (*n* = 5): [44, 48, 61, 71, 72].
	*Multiple benchmarks* (multiple groups or values, or individual peer scores) facilitates feedback acceptance by increasing credibility because it helps recipients assess variation between professionals and judge whether potential discrepancies are clinically significant.	Theories (*n* = 2): Feedback Intervention Theory [7], Social Comparison Theory [31]. Qualitative studies (*n* = 6): [37, 40, 57, 59, 73, 74].
	*Multiple benchmarks* allow recipients to make downward social comparisons (defensive response to feel better about themselves) instead of upward social comparisons which inhibit action.	Theory: Social Comparison Theory [31].
Trends	Facilitates action by decreasing the complexity in a way that helps recipients interpret and identify when clinical performance requires action, in particular, if the *reference period* includes sufficient time points at regular intervals dependent on the performance topic and number of observations each interval.	Theories (*n* = 1): Feedback Intervention Theory [7]. Qualitative studies (*n* = 11): [37–39, 44, 46, 50, 51, 55, 77–83].
	Increases the observability of the feedback intervention which induces positive emotions by demonstrating how recipients’ clinical performance has improved over time as a consequence of their taken actions; higher improvement rates being associated with higher satisfaction.	Theories (*n* = 2): Feedback Intervention Theory [7], Johnson et al. [30]. Qualitative studies (*n* = 7): [44–46, 77–80].
	Facilitates acceptance of feedback by increasing its credibility because performance is measured during a *reference period* that includes multiple time points (e.g. to eliminate the possibility of one-time coincidentally low performance).	Qualitative studies (*n* = 2): [39, 45].
Explicit targets	Facilitates action by reducing complexity of the feedback, making it easier for recipients to know what constitutes ‘good performance’ and therefore what requires a corrective response.	Theories (*n* = 3): Control Theory [9], Goal-setting Theory [13], Feedback Intervention Theory [7]. Qualitative studies (*n* = 2): [84, 85].
	Targets from an external *source* that lacks power or credibility inhibit acceptance of negative feedback by inducing creates cognitive dissonance; recipients may respond by rejecting the target/feedback to resolve this dissonance and maintain the perception of self-integrity, rather than question their own competency as a clinician.	Theories (*n* = 4): Ilgen et al. [25], Cabana et al. [26], Theory of Cognitive Dissonance [28], Self-affirmation Theory [29]. Qualitative studies (*n* = 2): [68, 84].
	Self-set targets (i.e. *source* is feedback recipients themselves) increase goal commitment and progress towards the target, but recipients may choose inappropriate targets (i.e. too low or unachievably high) to eliminate the discrepancy or because they do not know how to set targets.	Theories (*n* = 1): Goal-setting Theory [13]. Qualitative studies (*n* = 2): [85, 86].
	Ambitious target *values* increase feedback effectiveness over simple targets as long as they are (considered) achievable.	Theories (*n* = 2): Goal-setting Theory [13], Feedback Intervention Theory [7].
	Absolute target *values* are simple (decreasing complexity) than relative targets but can become outdated when achieved by most recipients which inhibits continuous quality improvement.	Theories (*n* = 1): Control Theory [9].
	Relative targets based on benchmarking facilitate continuous quality improvement as can be automatically adjusted when the group performance changes, but also inhibits action because it creates uncertainty to recipients as to which performance levels should be targeted.	Qualitative studies (*n* = 1): [72].
	Relative target *values* based on benchmarking inhibit feedback acceptance if recipients consider them unfair, in particular, if performance is just below target and variation between peers is small and clinically insignificant.	Qualitative studies (*n* = 2): [59, 84].

**Table 3:** Performance comparators used in the 146 included audit and feedback interventions

Performance comparators	*n* (%)
Benchmarks	88 (60.3)
Reference group	
Region	39 (24.7)
State or province	26 (17.8)
Country	21 (14.4)
Unit or department, e.g. individual physicians within a hospital	12 (8.2)
Multistate	5 (3.4)
Same type units, e.g. teaching hospitals	3 (2.1)
Other: city or small group	4 (2.7)
Values	
Mean	37 (25.3)
Individual peer scores—anonymous or unclear if identifiable	23 (15.8)
Top 10% mean (or ABC benchmark^a^)	7 (4.8)
Median	6 (4.1)
Other percentiles, e.g. 75th or 80th percentile	6 (4.1)
Rank or percentile rank	4 (2.7)
Individual peer scores—identifiable	3 (2.1)
Other, e.g. min-max or standard deviation	3 (2.1)
Unclear	22 (15.1)
Trends	17 (9.6)
Reference period	
Previous 1–6 quarters	7 (4.8)
Previous 1–12 months	4 (2.7)
Previous 1–6 half years	2 (1.4)
Previous 1–15 weeks	2 (1.4)
Previous 1 year	1 (0.7)
Unclear	1 (0.7)
Explicit targets	16 (11.0)
Source	
Investigators	5 (3.4)
Feedback recipients or local management (i.e. self-set targets)	5 (3.4)
Expert panel	3 (2.1)
Other: government or guideline	3 (2.1)
Unclear	1 (0.7)
Values	
Absolute targets, e.g. 80% performance level	6 (4.1)
Relative targets based on benchmarking, e.g. 80th percentile of baseline peer performance	6 (4.1)
Relative targets based on trends, e.g. 20% improvement from baseline	3 (2.1)
Unclear	1 (0.7)
No comparators or unclear	48 (32.9)

Items are not mutually exclusive

^a^*ABC benchmark* achievable benchmark of care, defined as the mean performance level achieved by the top 10% [64]

### Benchmarks

In 88 (60.3%) interventions, the feedback included benchmarks, i.e. comparisons of recipients’ achieved performance against that of other health professionals or peers. Benchmarks could be characterised by the group of peers being compared against (*reference group*), and the group’s performance was represented (*summary statistic*). We identified 7 theories, 5 trials, and 32 qualitative studies that suggested mechanisms relevant to benchmarking (Table 2). Although benchmarks in principle do not necessarily explicitly state what levels recipients are expected to achieve, they may be perceived as targets that recipients use for improvement. In fact, they can harness competition between recipients (Social Comparison Theory [31]) and motivate recipients to change behaviour if they see others behaving differently (Persuasion Theory [27] and Social Norms Theory [33]), trying to maintain their status in a group of high-performing clinicians (Reference Group Theory [32]). Recipients who observe that others are achieving a certain level of performance may find it easier to conceive that they can too. While a wide array of qualitative studies support these theoretical mechanisms [34–52], Feedback Intervention Theory [7] counters that benchmarking debilitates the effects of feedback by directing recipients’ attention away from the task at hand (i.e. the clinical performance issue in question, such as prescribing appropriate medication). Two trials comparing feedback with versus without benchmarks, however, both found small increases in effectiveness [53, 54]. Qualitative studies showed furthermore that benchmarks induced positive emotions (e.g. reassurance, satisfaction) when recipients observed they were performing better than or similar to others [39, 49, 55–59], or negative emotions (e.g. embarrassment) and consequent feedback rejection when recipients performed at the lower end of the distribution [49, 58]. In 1 A&F trial, involving an intervention to increase use of a preferred drug, Schectman et al. [60] explicitly chose not to include benchmarks because they expected it to discourage greater use because overall use was low.

#### Reference group

Benchmarks were typically drawn from the performance of peers in the same region (*n* = 39; 24.7%), state or province (*n* = 26; 17.8%), country (*n* = 21; 14.4%), or—in case of individualised feedback—other health professionals within the same unit, hospital, or department (*n* = 12; 8.2%). In 3 (2.1%) cases, benchmarks concerned similar-type peers such as only teaching hospitals or non-teaching hospitals. Finally, in 19 (13.0%) cases, comparisons to multiple peer groups were provided, such as the region and country, or only teaching hospitals and all hospitals in the province. Qualitative studies reported that recipients were more likely to accept the benchmark when they considered its reference group relevant and comparable [36, 39, 40, 51, 52, 61–63], as also hypothesised by the Reference Group Theory [32]. This suggests that regional comparisons are typically preferred over national ones, and comparisons that differentiate between the type of peers may be more effective than those that do not. Alternatively, recipients rejected feedback when they felt that the comparison was irrelevant or unfair, such as when they perceived inadequate case-mix adjustment or patient stratification [36, 39, 52, 62, 63].

#### Summary statistic

The most common benchmark value was the group mean (*n* = 37; 25.3%). Other summary statistics used were the mean of the top 10% peers (*n* = 7; 4.8%; also known as the achievable benchmark of care, or ABC benchmark, defined as the mean performance achieved by the top 10% best performers of the group [64]), the median (*n* = 6; 4.1%) or various other percentiles such as the 75th or 80th percentile (*n* = 6; 4.1%), and the recipient’s rank or percentile rank in the group (*n* = 4; 2.7%). In contrast to using a summary statistic as the value of a benchmark, feedback in 26 (17.8%) interventions presented the individual performance scores achieved by peers in the group, e.g. in a bar chart, histogram, or table. Twenty-two (15.1%) times, it was not reported or unclear how peer performance was represented. Despite the mean being the most popular choice, others have used higher levels, e.g. the 80th percentile or top 10% of peers, as these could more clearly demonstrate discrepancies between actual and desired performance for the majority of feedback recipients [65–67]. Benchmarking against the mean reveals such discrepancies to at most half of the recipients and may not lead to the desired intentions to achieve the highest standards of care (Control Theory [9]). This was also supported by several qualitative studies in which recipients were not prompted to improve because the performance was ‘in the middle’ [35, 59, 68], or recipients were dissatisfied by comparing against the mean because they did not consider it as being the gold standard [35, 62]. In a randomised trial comparing two variations of benchmarks, Kiefe et al. [65] found that comparing to the top 10% of peers led to larger feedback effectiveness than comparing to the mean. However, Schneider et al. [66] found that identifying the top performers in the context of a quality circle did not improve the effectiveness of feedback. Consistent with Goal-setting Theory [13], some low performers considered such high benchmarks unachievable and questioned or disengaged from the feedback [35, 62] and may have benefitted more from comparing to the mean.

Feedback in three (2.1%) interventions presented individual peers’ performance scores while making the identities of those peers visible to recipients. In two cases, this concerned all peers [69, 70], whereas the other, only the top performer was identified [66]. This approach may be effective as it allows recipients to choose the most relevant peers for comparison (Reference Group Theory [32]) and further increases their sense of competition knowing that their own performance is reported to others (Social Comparison Theory [31]). However, qualitative studies have reported that recipients experienced such open reporting as threatening and therefore preferred anonymous data [44, 48, 61, 71, 72].

#### Multiple benchmarks

Sixteen (11.0%) interventions used a combination of benchmarks, such as the mean and standard deviation, median and the top 10%, or peers’ individual scores and interquartile range. Several qualitative studies have indicated that providing multiple benchmarks (that is, against multiple groups, multiple summary statistics, or peers’ individual performance scores) may facilitate the credibility of feedback because it helps recipients assess variation between professionals and judge whether potential discrepancies are clinically significant [37, 40, 57, 59, 73, 74]. However, it also increases the complexity of the feedback message—making it more difficult to understand whether performance requires attention or not as there are multiple values to which recipients can compare (Feedback Intervention Theory [7]). This allows recipients to make downward social comparisons, a defensive tendency in which they compare themselves against a group or individual that they consider ‘worse off’ in order to make themselves feel better about themselves (Social Comparison Theory [31]). In contrast, recipients who compare themselves against a group or individual that they perceive as superior can facilitate self-evaluation and improvement [31].

### Trends

Feedback in 17 (9.6%) interventions included trends, i.e. comparisons to recipients’ own previously achieved performance over a specified period (*reference period*). We identified 2 theories and 12 qualitative studies that suggested mechanisms relevant to trends (Table 2). For example, Foster et al. [75] provided 1-time feedback at 6 months after the start of a multifaceted educational programme to increase adherence to asthma guidelines in which recipients’ current performance was compared to that at baseline. Rantz et al. [76] provided feedback that included trends displayed as a line graph of recipients’ performance over the previous 5 quarters. Trends allow recipients to monitor themselves and assess the rate of change in their performance over time. Feedback Intervention Theory [7] and theory on self-regulation [30] refer to this as *velocity feedback* and indicate that rapid rates of improvement lead to more goal achievement and satisfaction, whereas constant or delayed improvement rates ultimately lead to withdrawal. Empirical studies found that recipients who observed deteriorating performance were often prompted to take corrective action [37–39, 44, 46, 50, 51, 55, 77–83]. Upward trends made successful change observable to recipients which promoted satisfaction and other positive emotions [44–46, 77–80]. Feedback messages that include performance at multiple time points may also facilitate the credibility of the message if a single instance of low current performance would have been considered a ‘snap shot’ explained away as chance or seasonal effects [39, 45]. However, past performance does not clearly guide improvement: it tells recipients where they came from but not where they should end up. This may be 1 of the reasons that 13 of the 17 studies provided additional comparators (benchmarks or explicit targets).

#### Reference period

The reference period used to display trends, described by the number of time points and intervals of past performance, was typically consistent with the number of times and frequency with which feedback was provided. Most often, trends displayed quarterly (*n* = 7; 4.8%) or monthly (*n* = 4; 2.7%) performance; other variants were weekly (*n* = 2; 1.4%), biyearly (*n* = 2; 1.4%), or yearly (*n* = 1; 0.7%). While qualitative studies reported that recipients valued ‘regular updates’, the exact frequency preferred by recipients typically depended on the clinical topic and the number of observations (e.g. patients) available each audit [37, 39, 45, 46, 82, 83].

### Explicit targets

In 16 (11.0%) interventions, health professionals received feedback with an explicit target: a specific level of achievement that is explicitly expected. Targets could be characterised by the person or party setting the target (*source*) and the level it is set at (*value*). Seven theories and 6 qualitative studies suggested mechanisms relevant to targets (Table 2). The use of explicit targets reduces the complexity of feedback messages because it makes it easier for recipients to know what needs to be attained and whether corrective response is necessary (Control Theory [9], Goal-setting Theory [13], Feedback Intervention Theory [7]). Two qualitative studies confirmed this [84, 85]. Explicit targets can be based on expert opinion, healthcare policies, performance data (e.g. benchmarks or trends), or a combination of these. The main difference between explicit targets, benchmarks, and trends is that the latter 2, despite potentially revealing important discrepancies with desired practice, may not explicitly judge current performance, leaving it to recipients to determine whether their performance is acceptable or not.

#### Source

Targets were set by an external party (i.e. *externally set targets*; *n* = 11) or locally by feedback recipients themselves (i.e. *self-set targets*; *n* = 5); two interventions used both. External targets were set by an expert panel (*n* = 3; 2.1%), investigators (*n* = 5; 3.4%), or guidelines or government (*n* = 3; 2.1%). Once (0.7%) it was unclear. While powerful target-setting sources can influence recipients’ decisions to take action, theory by Ilgen et al. [25] predicts that feedback from a source with low power or credibility is easily rejected. Cabana’s model of guideline adherence [26] indicates that physicians may have various reasons for non-adherence to recommended target, such as disagreement or lack of self-efficacy or outcome expectancy. Accepting a message indicating that performance is below a target requires recipients to acknowledge the fact that they are underperforming. However, this might conflict with the self-perception of being a capable and competent health professional, a situation referred to as cognitive dissonance (Theory of Cognitive Dissonance [28]). The theory states that recipients might find it easier to resolve this conflict by rejecting the externally imposed target, rather than question their own competency—even if the feedback holds compelling and meaningful information. Two qualitative studies reported similar response by recipients due to cognitive dissonance [68, 84]. Self-affirmation Theory [29] explains that such defensive responses arise, in part, from the motivation to maintain self-integrity. Affirmations of alternative domains of self-worth unrelated to the provoking threat (e.g. by also emphasising on high performance on other care aspects) can help recipients deal with threatening information without resorting to defensive response [29].

When feedback recipients set performance targets themselves (*self-set targets*), they are more likely to commit to and gain progress towards the targets (Goal-setting Theory [13]). Qualitative studies have shown that feedback with self-set targets may decrease the consistency in clinical performance across recipients [85, 86], in particular, if they are not supported by an external information source (e.g. benchmarking). Furthermore, recipients might adapt their targets to performance to eliminate discrepancies rather than vice versa (Feedback Intervention Theory [7]).

#### Values

Ambitious targets are more effective than easy ones as long as they are achievable (Goal-setting Theory [13] and Feedback Intervention Theory [7]). However, it might prove difficult to define a single target that is perceived as both ambitious and achievable by all recipients of a feedback intervention. Six (4.1%) interventions used absolute targets, or criterion-referenced targets, which are typically determined at or before baseline and do not change over time. For example, in Sommers et al. [87], an expert panel set a specific target (between 80 and 90%) for each quality indicator. Rantz et al. [76] provided 2 explicit targets to distinguish between good and excellent performance (e.g. 16% vs 6% rate of falls). In another 6 (4.1%) interventions, the targets related to benchmarking against best practice. For example, in Goff et al. [88], researchers set explicit targets at the 80th percentile of participants’ baseline performance. Finally, 3 (2.1%) interventions set targets based on trends. For example, Fairbrother et al. [89] awarded financial bonuses to recipients who achieved 20% improvement from baseline, and Curran et al. [90] fed back statistical process control charts with control limits depended by the unit’s past performance to define out-of-control performance. With absolute targets, it is possible for all recipients to pass or fail depending on their achieved performance level, whereas with relative targets by definition, discrepancies are only presented to a subset of recipients. Relative targets based on benchmarking may be considered unfair by recipients performing just below them, in particular when the distribution of performance scores is narrow and differences between health professionals are clinically insignificant [59, 84]. Incremental targets demonstrate discrepancies to all recipients but may be unachievable when baseline performance is already high. Absolute targets are very simple to understand, but can become outdated when achieved by most recipients and should be reset in response to changing performance levels to remain appropriate [91]. Relative targets based on benchmarking can be automatically adjusted when the provider group performance changes. This facilitates continuous quality improvement (i.e. targets increase as the group improves), but due to its changing nature, it also creates uncertainty to recipients as to which performance levels should be targeted to guide improvement efforts [72]. However, in the included studies, relative targets were all set once and did not change.

## Discussion

In an effort to inform the design and delivery of more reliably effective A&F, we reviewed 146 randomised trials to identify choices for delivering clinical performance comparators. Ninety-eight (67.1%) included 1 or more comparators. Health professionals’ performance was compared against the performance of others (*benchmarks*; 60.3%), the recipient’s own historical performance (*trends*; 9.6%), expected standards of achievement (*explicit targets*; 11.0%), or a combination of these (13.0%). Only 8 trials (5.5%) stated a rationale for using the specific comparators. We identified 12 behavioural theories and evidence from 5 randomised trials and 42 qualitative studies from which we distilled explanations of the mechanisms through which different comparators may support quality improvement.

### Comparison to existing literature

In a re-analysis of the earlier Cochrane review by Jamtvedt et al. [92] (118 trials), Hysong [93] found no effect of adding benchmarks to A&F, regardless of whether or not identities of peers were known to recipients. While our findings suggest that benchmarking should increase the effectiveness of A&F by harnessing the social dynamics between recipients, there remain unanswered questions with respect to how benchmarks could work best. In line with our results, two empirical studies of A&F [14, 15] demonstrated that benchmarking against the mean and the top 10% of performers influences recipients’ intentions to improve on quality indicators, even though these intentions are not always translated into effective action [94, 95]. Still, study participants ignored some benchmarks because they were too high or the indicator lacked priority [14].

The effect of explicit targets has been previously investigated by Gardner et al. [96] in their re-analysis of the Jamtvedt review [92]. Gardner’s results were inconclusive at the time because very few studies explicitly described their use of targets, but the 2012 update of the review [1] showed that target setting, in particular in combination with action planning, increased the effectiveness of A&F. The role of involving recipients in setting targets themselves remains uncertain in healthcare settings [97, 98]. An empirical study [15] showed that recipients may set their targets regardless of any benchmarks or trends and—potentially unrealistically—high, even when confronted with benchmarks of the top 10% reflecting much lower standards [15].

Brehaut et al. [5] recently advocated a single comparator that effectively communicates the key message. While multiple comparators may indeed send complex and mixed messages to recipients, we found that well-considered and presented multiple comparators may be beneficial to the effectiveness of A&F [99]. This underlines the complexity of this area and the need for more research.

### Implications for practice and research

Our findings are useful for guiding the design of A&F interventions with respect to choice of performance comparator in feedback messages. We have identified a wide variety of comparators that may be included in feedback messages, as well as mechanisms and outcomes that potentially occur as a consequence of those comparators in terms of what message the feedback conveys (i.e. whether and how it reflects discrepancies with desirable practice), how recipients might respond, and ultimately the effectiveness of A&F. Many of the mechanisms we identified originate from behavioural science which offers a great amount of theoretical and empirical evidence not often taken into account by feedback designers [4, 17]. The exact way in which a comparator modifies that response and the intervention effectiveness depends on various factors relating to the individual recipient or team, organisation, patient population, and/or clinical performance topic, in addition to whether/how the comparator reveals a discrepancy with current practice [19]. A&F designers should explicitly consider these factors and the mechanisms we presented and offer justification for their choice of comparator.

A single type of comparator that works for all recipients and for all care processes or outcomes targeted by the A&F intervention may not exist. Comparators should be designed to maximise feedback acceptance in the context of raising standards of care via multiple means. Based on our findings, we have four suggestions for choosing comparators:

1. Step away from benchmarking against the mean and consider tailored performance comparisons

Benchmarks work by leveraging the social dynamics between recipients, the main mechanisms of which have been described by the Social Comparison Theory [31] and Reference Group Theory [32]. However, 42% of the A&F interventions included in this study that used benchmarking involved comparisons to the group mean. The theory predicts, and qualitative and quantitative evidence have demonstrated, that such comparisons are unlikely to raise performance levels comprehensively across feedback recipients. We recommended that recipients compare themselves to high-performing others that are both relevant and comparable to the recipient. However, if benchmarks are too high, they may be perceived as unachievable for low performers and lead to feedback rejection, or other unintended consequences. For example, a recent A&F study to reduce high-risk prescribing in nursing homes felt that benchmarking against the top 10% may risk unintended discontinuation of appropriate medications and therefore compared against the top quartile instead [100]. A solution to this problem may lie in tailoring of feedback messages to individual recipients or practices [12], for example by comparing low performers to the mean or median and others to the top 10%.

2. Balance the credibility and actionability of the feedback message

Qualitative studies have found feedback credibility and actionability to be important characteristics that should be properly balanced when choosing comparators. Based on a single comparator, health professionals may explain negative feedback away as a coincidental ‘snapshot’ of low performance, or question the data quality or fairness of the comparison [101]. Offering multiple performance comparators may help recipients assess whether there are true discrepancies with desired practice. For example, trends reveal whether low performance was one-time or has been consistent over time, and multiple benchmarks (e.g. individual peer scores) indicate performance in light of the variation between health professionals. Although providing multiple comparators may therefore increase the credibility of the feedback, it also increases its complexity and cognitive load and might send mixed messages to recipients. For example, if a health professional’s performance has improved over time but remains below the top 10% of practices, a feedback message suggesting that improvement is needed might be inconsistent with the professional’s interpretation that ‘the numbers are improving so no further change is necessary’ [5]. Hence, feedback should be presented in a way that clearly presents the key message (i.e. improvement is recommended or not), limiting the amount of information (e.g. comparators) presented to increase actionability, while allowing recipients to view more detailed comparative information if desired to increase credibility.

3. Provide performance trends, but not trends alone

Trends enable recipients to monitor performance and progress over multiple time points, and many qualitative studies have shown that recipients likely act upon observed performance changes. In fact, Feedback Intervention Theory [7] and theory on self-regulation [30] show that the rate of performance change (i.e. velocity) may be a more important motivator for change than the distance between performance and a goal (i.e. discrepancy). Trends also increase the credibility of feedback and enable a quality improvement cycle in which recipients continuously self-assess their performance upon which they decide whether or not to act. Trends therefore add substantial value to feedback and should be an explicit part of feedback messages. However, since trends only provide information about performance of the past and not the goal, they should be accompanied with other comparators (i.e. a benchmark or explicit target) that provide explicit direction for further improvement.

4. Encourage feedback recipients to set personal, explicit targets guided by relevant information

Goal-setting Theory [13], and various theories that extend it, predicts that explicit targets reduce feedback complexity because they set specific, measurable goals. However, qualitative studies report that unless such externally set targets were set by a broadly recognised, credible authority (e.g. national guidelines) or are linked to financial incentives, accreditation, or penalties, they may not be acceptable for a subset of recipients. We therefore recommend that feedback recipients are encouraged to set their own targets, guided by relevant information drawn from guidelines, expert opinion, and performance data, to which explicit comparisons can be made in the feedback. Feedback providers can collaborate with recipients to ensure the appropriateness of targets. Although recipients may consequently pursue different targets, it also enables them to commit to self-chosen targets that are both achievable and appropriate for themselves which reduces the chance of feedback rejection.

### Strengths and limitations

To our knowledge, we are the first to have systematically considered existing relevant theories and empirical evidence to fill a key knowledge gap with regard to the use of clinical performance comparators in A&F interventions [4, 6]. Few past studies have explicitly built on extant theory and previous research [17]. This work helps advance the science in the field by summarising the practical considerations for the comparator choice in the A&F design.

There are also several limitations. In using the 2012 Cochrane review of A&F and 2017 systematic review of electronic A&F to identify current choices for performance comparators, we were limited to randomised controlled trials being evaluated in a research setting only. Other study designs, and A&F used in non-research routine healthcare settings, might have yielded other types and/or frequencies of performance comparators that have been used. In particular, because A&F in research settings likely emphasises performance improvement while routine A&F may focus more on performance monitoring, we expect that the comparators and mechanisms we identified are more aimed at activating recipients to improve practice, rather than only supporting recipients to assess their performance. Another limitation is the quality of reporting and lack of consistency with regard to the terminology for comparators, particularly in the older studies [11, 102]. One way in which this particularly might have manifested is that it was often unclear to which extent performance comparators were delivered as explicit targets. For example, studies that have used a particular benchmark may have added an explicit message that they are expected to achieve that standard, making the benchmark an explicit target as well, but it has not been reported as such in the paper. As a result, despite the prominence of targets in existing feedback theories [7, 9, 13], we have found limited evidence about the use of explicit targets.

Our review was limited to performance comparators at an aggregated level. When feedback is provided about individual patient cases, comparators at the patient-level may be included which allow feedback recipients to make performance comparisons for each patient [103]. We also did not explore the influence of the way in which comparators were displayed or represented in the feedback messages. Finally, we did not use meta-regression to examine and quantify the effects of each comparator because such an analysis would be vastly underpowered as a result of the large variety in comparator use across trials.

### Unanswered questions and future research

Colquhoun et al. have generated a list of 313 theory-informed hypotheses that suggest conditions for more effective interventions of which 26 related to the comparators [6]. Our research delivers some important pieces of the puzzle to design and deliver effective A&F, but many other pieces are still missing. To move the science forward, more of these hypotheses should be tested. Within the domain of performance comparators, theory-informed head-to-head trials comparing different types of comparators (e.g. [100, 104]) are needed to help uncover successful comparators tested under similar conditions.

## Conclusion

Published A&F interventions have typically used benchmarks, historic trends, and explicit targets as performance comparators. The choice of comparator seemed rarely motivated by theory or evidence, even though abundant literature about feedback mechanisms exists in theories from behavioural and social sciences and empirical studies. Most interventions benchmarked against mean performance which is unlikely to comprehensively raise the standards of care. There appears to be considerable opportunity to design better performance comparators to increase the effectiveness of A&F. Designers of A&F interventions need to explicitly consider the mechanisms of comparators and offer justification for their choice.

## Additional files

Additional file 1: Identifying behaviour change theories (DOCX 175 kb)

## Abbreviation

A&F: Audit and feedback
